# Interactions between Beer Compounds and Human Salivary Proteins: Insights toward Astringency and Bitterness Perception

**DOI:** 10.3390/molecules28062522

**Published:** 2023-03-09

**Authors:** Leonor Gonçalves, Mónica Jesus, Elsa Brandão, Paulo Magalhães, Nuno Mateus, Victor de Freitas, Susana Soares

**Affiliations:** 1 Faculdade de Ciências, Universidade do Porto, Rua do Campo Alegre, 689, 4169-007 Porto, Portugal; 2 REQUIMTE/LAQV, Faculdade de Ciências, Universidade do Porto, Rua do Campo Alegre, 689, 4169-007 Porto, Portugal; 3 Super Bock Group, S.A., Via Norte, 4465-764 Leça do Balio, Portugal

**Keywords:** beer, astringency, bitterness, salivary proteins, phenolic compounds

## Abstract

Beer is one of the most consumed beverages worldwide with unique organoleptic properties. Bitterness and astringency are well-known key features and, when perceived with high intensity, could lead to beer rejection. Most studies on beer astringency and bitterness use sensory assays and fail to study the molecular events that occur inside the oral cavity responsible for those perceptions. This work focused on deepening this knowledge based on the interaction of salivary proteins (SP) and beer phenolic compounds (PCs) and their effect toward these two sensory attributes. The astringency and bitterness of four different beers were assessed by a sensory panel and were coupled to the study of the SP changes and PC profile characterization of beers. The human SP content was measured before (basal) and after each beer intake using HPLC analysis. The beers’ PC content and profile were determined using Folin–Ciocalteu and LC-MS spectrometry, respectively. The results revealed a positive correlation between PCs and astringency and bitterness and a negative correlation between SP changes and these taste modalities. Overall, the results revealed that beers with higher PC content (AAL and IPA) are more astringent and bitter than beers with a lower PC content (HL and SBO). The correlation results suggested that an increase in whole SP content, under stimulation, should decrease astringency and bitterness perception. No correlation was found between the changes in specific families of SP and astringency and bitterness perception.

## 1. Introduction

Beer is one of the most popular alcoholic beverages consumed in the world. However, consumers’ preferences and choices differ, especially in the basic taste properties of beer (e.g., bitterness, sweetness, and sourness) and mouthfeel (e.g., astringency and fizziness) [1]. Although beer is commonly brewed using four traditional ingredients (malt, hops, yeast, and water), it is a natural, outstanding complex beverage made of carbohydrates, minerals, vitamins, lipids, protein/amino acids, and phenolic compounds (PCs) [1,2,3]. In fact, hops (*Humulus lupulus* L.) are the major contributor to bitter taste perception in beer [4]. Moreover, some amino acids (e.g., L-histidine and L-tryptophan) [5,6,7], lipids (e.g., linoleic, and oleic acid) [8], and phenolic compounds (e.g., catechin and epicatechin) [9,10,11] are also known for their ability to taste bitter and/or astringent.

Therefore, bitter compounds are present in different types of beer and have been identified and quantified with high-performance liquid chromatography with diode array Detection (HPLC-DAD) and mass spectrometry. Although humans have innate adversity to bitter compounds, beer bitter compounds are in part responsible for the refreshing taste in all brewed beer brands [4] and certain beers′ taste characteristics may benefit from having a strong bitterness, but not all of them. In fact, the bitter attributes of beer depend on the diversity and quantity of compounds, which in turn depend on the hops’ adding time and brewing conditions. Hops possess features of secondary metabolites, which are transformed over the brewing process into flavoring (e.g., α-humulone and β-caryophyllene) and bittering (e.g., α-acids, β-acids, and xanthohumol) components [4]. In addition to the naturally present bitter compounds, the bittering of beers is also usually applied by boiling hop α-acids, converting them into iso-α acids, which are bitter. Thus, not all the bitter compounds present in beer derive from hops [4].

Beer constitutes a good source of PCs, 15–25% of which hail from hops and 75–85% from malt [3,9]. These PCs are mainly flavonoids, as flavan-3-ols (e.g., catechin), iso-flavones (e.g., xanthohumol), and nonflavonoids such as phenolic acids (e.g., gallic acid) [4,12]. Additionally, hops contain monoacyl phloroglucides, which are transformed into bitter acids (humulones and lupulones) during the brewing process. Xanthohumol is essentially in the form of isoxanthohumol (22–30% of hop xanthohumol), and about 10% of desmethylxanthohumol is completely transformed into prenyl naringenin [4]. Quiffer and colleagues (2014) identified forty-seven PCs in beer, including flavanols, flavonols, flavones, simple phenolic acids, hydroxycinnamoylquinics, hydroxyphenylacetic acids, alkymethoxyphenols, alpha- and iso-alpha-acids, and prenylflavanoids [9]. Cortese et al. (2019) identified twenty PCs in different craft beers, and Cheiran et al. (2019) reported fifty-seven PCs, where twelve of them were found for the first time in beer [10,11]. Another set of PCs designated to have the ability to bind to proteins and to be linked to astringency is tannins, and they are divided into hydrolysable (e.g., ellagic and gallic acid) and condensed tannins (e.g., procyanidins) [13,14,15]. Condensed tannins, also referred to as proanthocyanidins, are made from flavanol units, namely catechin and epicatechin derivatives [16,17].

PCs attract special interest due to their health benefits by having antioxidant activity and preventing cardiovascular and neurodegenerative diseases [18,19,20,21]. Transversely, some of these compounds are responsible for several organoleptic features of plant-based foodstuffs such as color and taste properties, such as astringency and bitterness [22]. Regarding consumers’ preferences and choices, color is one of the first and most significant quality parameters in which a certain food product could be rejected by a depreciative visual perception [22]. In addition, astringency and bitterness are commonly perceived as unpleasant, but balanced levels between these two taste qualities are desired for beer consumption [22].

Astringency refers to a puckering, drying, and rough sensation in the oral cavity. The perception of astringency and bitterness taste properties occurs through different physiological mechanisms [22]. While bitter taste perception occurs through the activation of specific membrane G protein-coupled receptors, the bitter taste receptors (TAS2Rs), the molecular mechanism of astringency is still a debated topic [22,23]. The interaction and precipitation of salivary proteins (SPs), mainly proline-rich proteins (PRPs), by PCs is often believed as the major mechanism toward astringency perception [22,23,24], but more recently, the interaction with oral epithelial cells and the activation of trigeminal chemoreceptors has been also reported consistently [22,23,24,25].

The families of SPs that have been related to astringency include the proline-rich proteins (PRPs), namely basic PRPs (bPRPs), glycosylated PRPs (gPRPs), acidic PRPs (aPRPs), statherin, P-B peptide, cystatins, and mucins. These SP families present differences regarding to their amino acid residues and, therefore, their structure, their charge, and the presence or absence of carbohydrates [23,26]. PRPs are rich in proline residues and within this family, bPRPs are the richest in proline and glycine residues, being 70% of their sequence; gPRPs possess carbohydrates linked to some amino acid residues and aPRPs are rich in acidic amino acid residues such as glutamic acid (or glutamine). Statherin is an acidic SP rich in tyrosine residues, and P-B peptide is rich in proline residues (50% of its sequence) and is commonly linked to statherin because of gene proximity, but its structure shows some similarities with PRPs [26,27]. They have essential functions in saliva, such as the oral lubrication role by gPRPs and mucins, the homeostasis maintenance of calcium phosphate by statherin, and also oral tissue protection against degradation through inhibiting the cysteine protease activity by cystatins [23,26].

While SPs have been more related to astringency, recently, Martin et al. (2019) reported that SPs might also play a role in bitter taste, where SPs decrease the sensitivity to quinine, a bitter compound [28,29]. So, it is also important to study if there is a relation between the different groups of SP family and bitter taste perception.

Researchers have been attempting to find a relationship between instrumental and sensory data in order to better understand actual human perception. [30]. Few research papers could relate sensory data with instrumental data, and several correlations were reported between taste perception and instrumentally measured taste molecules [30]. Luna et al. (2002) established a positive correlation between PCs and the perception of bitterness, astringency, and green notes in cocoa samples [31].

Regarding beer, while there are data linking PC content to astringency and bitterness properties in humans [32,33,34], there is a lack of understanding about the molecular events involving the SPs inside the oral cavity that underpin these perceptions. So, this work is focused on deepening the knowledge on astringency and bitterness perception toward PCs’ interaction with SPs. To achieve this, this study was divided into two major aims: (i) to study the interaction of human SPs with beer *in vivo*, (ii) to characterize the variety of compounds (PCs and non-PCs) in different types of beer, and (iii) to identify any correlations between the studied instrumental variables (SPs and beer PC content) and the perceived astringency and bitterness. This work will provide more insights into the putative influence of these compounds on the taste properties of beer.

## 2. Results

In order to study the SP behavior within saliva upon beer intake and correlate it with the astringency and bitter attributes of each beer, a sensory analysis of four different beers was performed.

### 2.1. Sensory Analysis of Beers

A sensorial analysis was performed with an intern panel from the beer’s producer company (Super Bock Group).

For the sensory analysis, the panelists were asked to classify the four different beers as astringent and bitter according to a scale of 1 to 5, as referred to in Section 4.1.1. In Table 1, scale number 1 is represented by the symbol “+” standing for low astringency or bitterness, and scale number 5 is represented by “+++++” for high astringency and bitterness. The sensory analysis results showed that the panelists had different taste perceptions when perceiving the same type of beer. Moreover, the bitterness perception varied more between panelists than the astringency sensation, as there were beers where the intensity of bitterness perception was quite different, even when perceiving a similar intensity of astringency (e.g., HL). Furthermore, it was also evident that HL and SBO beers were less astringent and bitter than AAL and IPA beers.

### 2.2. Analysis of the Interaction between Beer and Salivary Proteins

**Figure 1:**
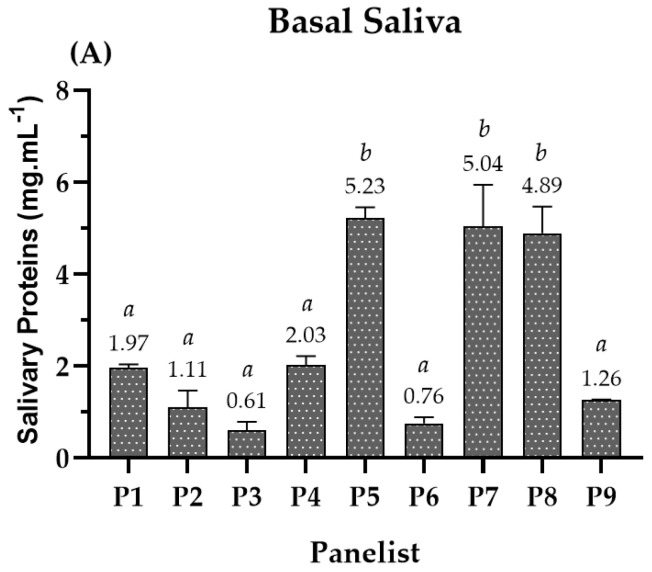

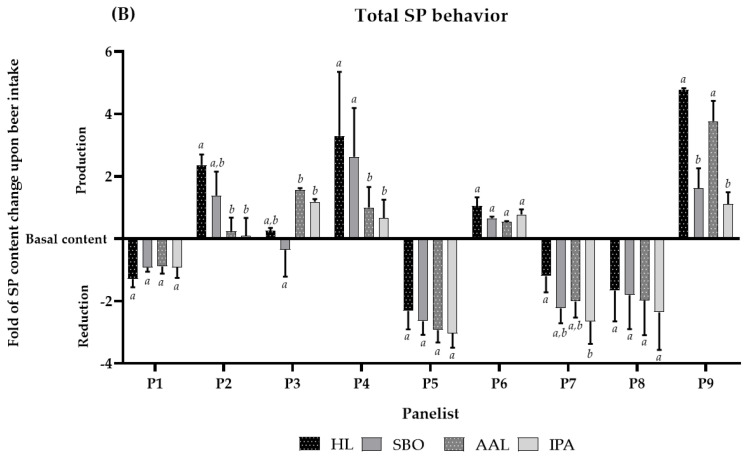
(**A**) Total concentration of soluble SP in basal saliva for each panelist. (**B**) SP behavior after intake of each beer, where Coruja Hoppy Lager (HL), Super Bock Original (SBO), Coruja American Amber Lager (AAL), and Coruja India Pale Ale (IPA), respectively. The SP behavior is presented by the fold of total soluble SP, for each panelist, during beer sensorial analysis. Fold represents how many times SP reduction or production occurs regarding the previous beer intake of saliva collection for each panelist. Values with different letters within each panelist are significantly different (*p* < 0.05).

A study involving *in vivo* interactions between beer (compounds) and human SP was coupled to the sensory analysis. This experimental approach intended to understand the two taste modalities’ perception (astringency, A; bitterness, B) at a molecular level. For this reason, basal saliva was collected from each panelist in unstimulated conditions before the sensory test. For that, saliva was accumulated and treated with trifluoracetic acid (TFA) to remove high-molecular-weight proteins and inhibit intrinsic protease activity for posterior HPLC analysis. Then, the total concentration of the soluble SP was determined in mg·mL^−1^ (Figure 1A) for each panelist. The results showed that the basal SP content varied among panelists, indicating that variability exists in SP production among the population. Regarding the range of SP content in basal saliva, it showed a variation between 0.6 and 5.5 mg·mL^−1^, where panelists 5, 7, and 8 had the highest SP content, approximately 5 mg·mL^−1^. The values obtained for the latest panelists were higher than the ones previously reported upon treatment with TFA (1.22 mg·mL^−1^) [28].

The panelists′ SP behavior after consuming beers was primarily divided into two different behaviors: SP content reduction in the basal level (e.g., panelist 1, 5, 7, and 8) or SP production due to saliva stimulation (e.g., panelist 2, 3, 4, 6, and 9). Interestingly, it was also observed that panelists with higher basal SP content (panelists 5, 7, and 8) showed a higher reduction pattern presented by a fold of 2 to 4x (e.g., panelist 5). In general, the panelists had statistically equal behavior and SP fold regardless of the studied beer (e.g., panelist 1, 4, 5, 6, and 8), reinforcing these results. So, these SP changes seem to be intrinsic to the panelists (Figure 1B).

**Figure 2:**
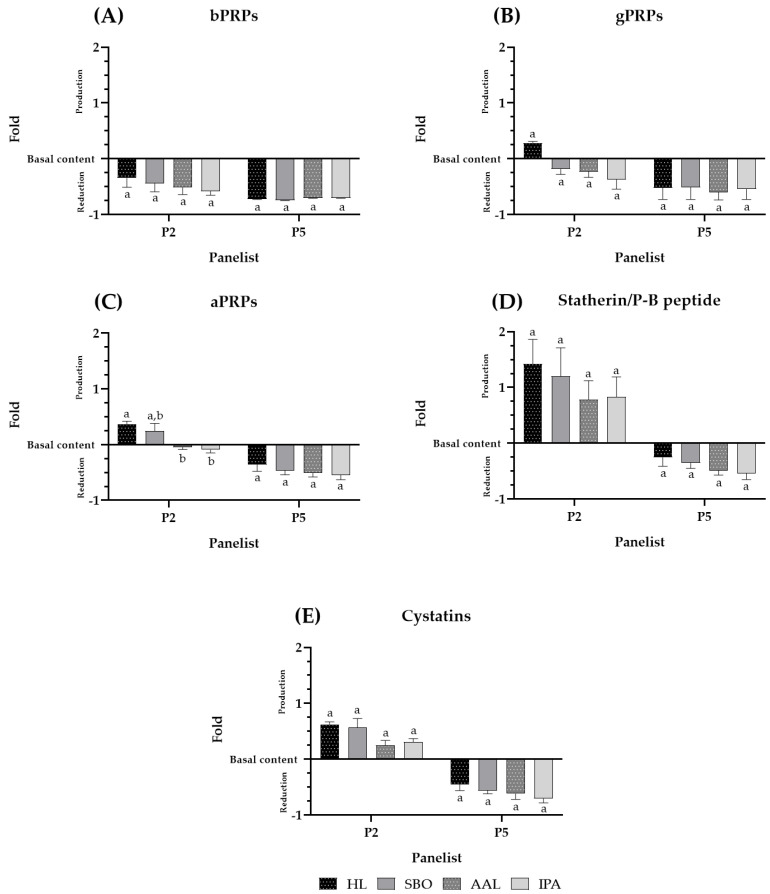
SP family behavior upon beer intake (Coruja Hoppy Lager (HL), Super Bock Original (SBO), Coruja American Amber Lager (AAL), and Coruja India Pale Ale (IPA), respectively. (**A**–**C**) indicate the PRP family, bPRPs, gPRPs, and aPRPs, respectively. (**D**) statherin and P-B peptide, and (**E**) cystatins upon beer intake. The interaction between the SP and PC compounds is presented by the fold of total SP precipitated, for each panelist, during beer sensorial analysis. Fold represents how many times SP reduction or production occurs regarding the previous beer intake of saliva collection for each panelist. Values with different letters within each panelist are significantly different (*p* < 0.05).

To observe more in detail what occurs with every single SP family quantified in the panelists′ saliva, Figure 2A–E show the SP family behavior upon each beer intake for two panelists, P2 and P5, as representatives of the panelists with SP above and below the basal level, respectively. The data from the other panelists are similar and presented in the Supplementary Materials (Figure S2).

The behavior of the different SP families appeared to be different among panelists, and these differences came from the different contribution of SPs to each panelist. Panelists 2 and 5 showed a total SP production and reduction pattern, respectively, upon beer intake (Figure 1B). However, the following analysis showed a similar tendency for all the PRP families (bPRPs, gPRPs, and aPRPs) but a distinct tendency for the statherin/P-B peptide and cystatin families for both panelists. In general, for both panelists, all PRPs were decreased by the same fold below their basal concentration, for panelist 2 (P2) statherin/P-B peptide, and cystatins occurred at a higher level than their basal concentration.

In order to quantify the phenolic content present in the studied beers, a solid-phase extraction (SPE) was performed to remove interfering compounds from the beers, and also to concentrate the compounds of interest in the sample (mainly phenolic compounds).

### 2.3. Total Phenolic Content Determination

The results of the phenolic content in the beer samples after solid-phase extraction (SPE) are shown in Table 2. The total phenolic content ranged from 0.40 to 1.0 mg·mL^−1^ [35].

According to general observation, HL and SBO had significantly lower phenolic contents than AAL and IPA.

### 2.4. Identification and Characterization of Beers

The identification and characterization of phenolic and nonphenolic compounds present in the analyzed beers were also performed with the SPE beer fractions. The total ion current (TIC) profile obtained using an LC-MS analysis of the four beers as well as the identification containing all the LC-MS information about the tentatively identified compounds present is shown in Figure S1A–H and Table S1 of Supplementary Materials, respectively. Sixty-two compounds were identified, of which twenty-seven were phenolic compounds (PC) and the remaining were nonphenolic compounds, such as amino acids, alkaloids, and fatty acids, among others. Table 3 and Table 4 indicate the compounds (phenolic and nonphenolic, respectively) identified in each beer and their taste quality according to the astringency and bitterness that have been already established. While some compounds were identified in all beers, others were not, most likely because they occur below the level of detection.

After the identification of the compounds and focusing on the PC (Table 3), it was possible to observe that AAL beer had more compounds in common with IPA beer than HL and SBO beers. All the four beers had ten PCs in common, which were (-)-epicatechin, 6-prenylnaringenin, 8-prenylnaringenin, 2-hydroxycinnamic acid, resveratrol, 7-hydroxycoumarin, diferuloylputrescine, esculin, and met-coumaroylagmatine hexoside. Curiously, 2-hydroxycinnamic acid, commonly known as umbelliferon, was described as both an astringent and a bitter compound. All the other compounds, except diferuloylputrescine and met-coumaroylagmatine hexoside, were described as bitter compounds.

In addition, other compounds besides PCs were described as tasting bitter and/or providing an astringent sensation. Table 4 shows the fifteen nonphenolic compounds putatively identified in the analyzed beers.

The analyzed beers possessed nine nonphenolic compounds in common: L-phenylalanine, L-tryptophan, pyroglutamyl-isoleucine, cyclo(proline-leucine), glutamyltyrosine, 8-hydroxyquinoline, caffeine, hordenine, and citric acid. In fact, L-phenylalanine, 8-hydroxyquinoline, caffeine, and hordenine were described as bitter compounds. L-tryptophan was astringent and bitter, and citric acid was astringent.

In order to observe the variability in the astringent and/or bitter compounds that were present in three to four beers, the chromatographic peak area values of each corresponding compound were determined. Seven of all the analyzed compounds showed greater variation among beers (Table 5). The results showed a greater amount for compounds described as bitter and/or astringent in AAL and IPA beers. AAL showed bitter compounds L-phenylalanine, (-)-epicatechin, and lupulone, and the astringent and bitter compound L-tryptophan; and IPA beer showed the bitter compounds (-)-epicatechin and lupulone, and the astringent and bitter compound L-tryptophan. Interestingly, L-phenylalanine was shown to be the most prevalent bitter compound in AAL beer, (-)-epicatechin in AAL and IPA beers, 8-prenylnaringenin in HL beer, and lupulone in IPA beer; L-tryptophan showed to be the most prevalent astringent and bitter compound in all the studied beers. These results suggest that all these compounds contribute to the bitter and or astringent perception of each beer, and that L-phenylalanine, (-)-epicatechin, lupulone, and L-tryptophan may be more abundant in AAL and IPA beers, resulting in more bitter beers, compared to HL and SBO beers, as verified by the sensory data.

### 2.5. Statistical Analysis

To identify any correlation between the sensory analysis (astringency and bitterness perception, Section 2.1) and the previously presented instrumental analysis (the beer compounds and SP changes in Section 2.2, Section 2.3, and Section 2.5), a statistical analysis was performed. The software XLSTAT version 2020.4.1.1020 was used to analyze the sensory attributes, astringency, and bitterness of four different types of beer by using multiple factor analysis (MFA) and sensory panel analysis.

**Figure 3:**
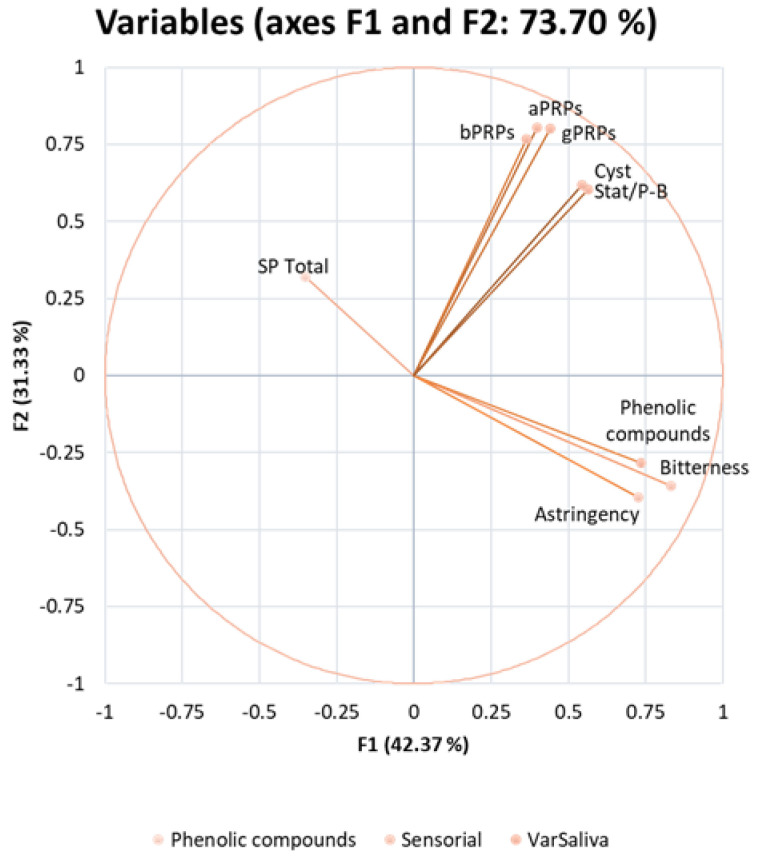
Multiple factor analysis (MFA) correlation circle of salivary proteins, phenolic compounds, and sensory attributes (sensorial), astringency, and bitterness after beer intake. Salivary proteins include bPRPs, gPRPs, aPRPs, statherin, and P-B peptide, cystatins, and total SPs, which comprise the total content of each previous SP family content, upon each beer intake (VarSaliva—variation in saliva).

The MFA was generated to study the correlation between the two sensory attributes and the different families of SPs and PCs. The first and second component explained 31.33% and 42.37% of the variation, respectively. Thus, the studied factors explained 73.70% of the total variation. The MFA correlation circle (Figure 3) was obtained to identify the possible correlations within the studied variables. Two positive correlations were observed: one between all SPs, with all PRPs being more closely related together and statherin/P-B peptide and cystatins also being closely related; the other was between PC and bitterness and astringency. A negative correlation was noted between the total SPs with bitterness and astringency, and there was no correlation between the different families of SPs and these two sensory perceptions.

## 3. Discussion

While astringency has been described as a mouthfeel sensation of puckering, roughness, and constriction in the oral cavity, its molecular perception has not been fully understood. Astringency has been mainly related to the interaction of salivary proteins, mainly proline-rich proteins (PRPs) and phenolic compounds, and, more recently, also through the interaction with oral epithelial cells, and the possible activation of mechanoreceptors (e.g., trigeminal chemoreceptors). These mechanisms could lead to a decrease in bitterness perception in the way that some of the bitter compounds are also astringent, and, therefore, would be “captured” in interactions with the oral constituents referred to previously and less available to interact with bitter taste receptors. In fact, this influence of astringency perception on bitterness has been already shown in mouse models [28].

Bitterness and astringency are two of the most essential beer qualities which are very important for consumers. According to the research from Da Costa Jardim and colleagues (2018), consumers tend to prefer beers which are less bitter [54].

The results obtained for bitterness perception (Table 1) correspond exactly with the organoleptic characteristics of these beer styles that have been established by the Super Bock Group. The four studied beers range from different styles and characteristics in terms of intensity, aroma, and flavor, being analyzed from the least to the most bitter beer.

The salivary protein profile of human saliva stabilized with TFA has been well characterized using HPLC analysis [13]. The SPs that remain soluble include the main families reported to be involved in astringency perception and to affect bitterness perception, namely PRPs. The behavior on the profile of the SP families (bPRPs, gPRPs, aPRPs, statherin/P-B peptide, and cystatins) was followed upon the interaction with beer compounds (Figure 1 and Figure 2). The behavior was followed before (basal saliva) and after beer intake.

From Figure 1A, it is possible to observe that the SP content is different within panelists. The total SP families content ranged from 0.6 to 5.5 mg·mL^−1^. Although the SP behavior upon beer intake was similar for a specific panelist, some panelists showed a reduction in the SP content below the basal level (e.g., P1, P5, P7, and P8) while others (e.g., P2, P3, P4, P6, and P9) showed an increase in SP above the basal level, indicating SP production upon beer intake throughout stimulation (Figure 1B). Interestingly, this SP production scenario occurred for the panelists that showed a lower basal SP content. According to Pedersen et al. (2018) [55], salivary production occurs under autonomic nervous control and is, hence, regulated by the sympathetic and parasympathetic nervous system. In particular, the stimulus arises from the activation of sensory receptors (e.g., chemoreceptors and mechanoreceptors) upon the ingestion of food and beverages, eliciting an increase in the production of protein-rich saliva and watery saliva, respectively [55]. This clearly justifies the production of saliva upon beer intake for some panelists. In addition, Condelli et al. (2006) observed that the intensity of astringency perception showed to be inversely related to saliva flow rate, and Linne and colleagues (2017) showed that subjects with low saliva flow had a higher sensory threshold for tannic acid [56,57].

Beers with a higher phenolic content possessed more astringency and bitterness compared to beers with a lower phenolic content. In fact, the total PC content in conventional beers was estimated to vary from 0.3 to 0.5 mg·mL^−1^. Higher values from 0.6 to 0.8 mg·mL^−1^ have been reported for bock and abbey beer types. Interestingly, Nardini and Foddai (2020) presented a study where the analyzed beers exhibited a PC content that ranged from 0.464 to 1.026 mg·mL^−1^ [58]. The beers analyzed in this study showed a total PC content in the range of 0.4 to 1.0 (Table 2), in accordance with the previously estimated values [32,59]. The statistical analysis revealed a positive correlation between PC content and bitterness and astringency. So, this result suggests that a beer with a higher PC content would be more bitter and/or astringent than a beer with a lower PC content. This is not a surprise because it is well known that PCs contribute significantly to astringency and bitterness perception. There are many studies that describe PCs as astringent and/or bitter, their interaction with salivary proteins (SP), and/or the activation of bitter taste receptors (TAS2Rs), respectively [13,14,16,52]. Indeed, Da Costa Jardim et al. (2018) showed that a less bitter beer typically may have a lower phenolic content [54]. Thus, HL and SBO beers were reported as less bitter and astringent, and they showed a lower PC content, compared to AAL and IPA.

Different beers should have different qualitative and quantitative compound profiles related to differences in the production method, among others; however, some compounds may be common across beers. The LC-MS analysis performed allowed us to putatively identify beer compounds (Table S1). Among the sixty-two compounds identified, twenty-seven were classified as PC and fifteen as nonphenolic compounds (Table 3 and Table 4), and some of these compounds are already established as astringent and/or bitter compounds [33,34,35,36,37,38,39,40,41,42,43,44,45,46,47,48,49,50]. Nineteen compounds were shown to be shared by all of the studied beers, but only thirteen PCs and ten non-PCs have already been described as astringent and/or bitter (Table 3 and Table 4). The variability in some of these PCs among all the beers (Table 5) suggests that (-)-epicatechin, lupulone L-phenylalanine, and L-tryptophan may contribute to the differences in the perception of the two taste modalities, as these compounds exhibited greater variability across the studied beers and also are present in the most bitter and astringent beers (e.g., AAL and IPA). (-)-Epicatechin, a bitter flavanol that activates TAS2R4, TAS2R5, and TAS2R39 [39], was shown to be in higher amounts in SBO beer. Lupulone, a hop bitter acid which activates TAS2R1 and TAS2R14 [47,60], showed to have higher levels in IPA beer. L-phenylalanine, a bitter amino acid that activates TAS2R1, was shown to be more abundant in AAL and IPA than HL and SBO beers [45,46]. L-tryptophan, another amino acid with astringent and bitter characteristics, has been described as a compound that activates TAS2R4 [5]. So, the previous PCs and amino acids may be four of the most prevalent compounds in these beers that could elicit the bitter and/or astringent taste sensation. In this study, AAL and IPA beers were found to have a higher total PC concentration (Table 2) and to be qualitatively richer in astringent and bitter compounds (Table 3 and Table 4). This is in agreement with the profile of the compounds presented previously for each beer.

In order to identify any correlation between the sensory analysis (astringency and bitterness perception) and the instrumental analysis, a statistical multiple factor analysis (MFA) was performed. The positive correlation between all families of SP is in accordance with the previous description regarding the different SP families, where PRPs belong to the same family, and statherin/P-B peptide and cystatins share few similarities. Another positive correlation was observed between PCs and bitterness and astringency, indicating that increasing the PC content of a sample will also increase the perception of bitterness and/or astringency, as expected. Curiously, a negative correlation was noted between the total SPs and bitterness and astringency, suggesting that a higher astringency and bitterness perception occurs when a decrease in the total SP content occurs. This negative correlation shows that the total SPs and these two taste modalities are inversely proportional. Interestingly, there was no correlation between any specific family of SPs and astringency or bitterness. While the changes in total SP content were directly linked to the changes in the different families of SPs, the changes in each family of SPs per se were not related to sensory perceptions.

This result is in line with our previous work that showed that, despite the prominence of some families of SPs in the saliva profile of the different individuals, the interaction with a specific PC-rich mixture occurred always in a similar way and was not affected by individual variability [59].

## 4. Materials and Methods

### 4.1. Sensory Analysis

#### 4.1.1. Sensory Panel

Beers were tasted by an intern panel of Super Bock Group (Porto, Portugal) composed of nine trained tasters. The panelists signed an intern informed consent form from Super Bock Group. Before sensory analysis, saliva from each panelist was collected upon 5 min of resting. Then, panelists were instructed to take a sip of the first beer, at room temperature, and saliva was collected after 5 min of accumulation. They had resting time (*ad libitum)* and performed a mouth rinse with water between beers before moving to the next beer. The volume of saliva collection ranged from 0.3 to 7.5 mL in these panel members. This procedure for astringency and bitterness sensory assessment has been already validated [61]. For the evaluation of astringency and bitterness intensity, the panelists scored from 1 to 5, with 1, 2, 3, 4, and 5 on the scale representing weak, moderate, strong, very strong, and extremely strong astringency and bitterness, respectively.

### 4.2. Experimental Procedures

For the instrumental evaluation of astringency and bitterness perception in beer, the saliva of each trained taster was straightaway collected before and after tasting each beer. First, basal saliva was collected before the tasting sessions to enable the measurement of baseline salivary protein content in each panelist. Then, saliva was collected after each panelist tasted and discarded each beer. Saliva was kept on ice until being transferred to microtubes. Each saliva sample was frozen on liquid nitrogen and kept at −80 °C until further analysis.

#### 4.2.1. Chemicals and Reagents

The acetonitrile (99.8%), methanol (99.8%), acetone (99.5%), acetic acid (99.7%), and trifluoroacetic acid (99.5%) were obtained from Sigma-Aldrich, St. Louis, MO, USA. Hydrochloric acid (37%) was from Fluka^®^, College Park, MD, USA. Chlorogenic acid (≥95%) was used as a phenolic standard and was purchased from Sigma-Aldrich, St. Louis, MO, USA.

#### 4.2.2. Saliva Treatment

Each saliva sample was prepared for high-performance liquid chromatography (HPLC) analysis. For the HPLC analysis, 10% trifluoroacetic acid (TFA, Sigma-Aldrich, St. Louis, MO, USA) was added to the saliva in a 1:90 *v/v* ratio. These samples were centrifuged at 8000× *g* for 5 min. The supernatant was analyzed.

##### HPLC Analysis

25 µL of each sample was analyzed on an HPLC Vanquish (Thermo Scientific, Waltham, MA, USA) equipped with an Agilent C8 column, with a 2.7 µm diameter and column dimensions of 4.6 × 150 mm, and detection was performed at 214 nm using a diode array detector (DAD). The HPLC solvents were 0.2% TFA in water (solvent A) and 0.2% TFA in acetonitrile/water 80/20 (*v*/*v*) (solvent B). A nonlinear gradient was applied as follows: 0 min, 15% B; 0–35 min, 46% B; 35–40 min, 55% B; 40–44 min, 90% B; 44–45 min, 100% B; 45–55 min, 100% B; 55–57 min, 15% B; and 57–67 min, 15% B, at a constant flow rate of 0.5 mL/min, and the column temperature was set at 25 °C. The peak areas were extracted at 214 nm and the total run time was 67 min. The samples were analyzed in triplicate and salivary protein concentrations were determined from calibration curves generated from external standards of PRPs, statherin, P-B peptide, and cystatins, in different ranges of mg·mL^−1^.

#### 4.2.3. Beer Samples

##### Beer Sample Preparation

Beer gas and foam were removed using ultrasonication coupled with a vacuum pump and, in addition, to maximize the degassing process, the samples were degassed with inert gas, such as argon. Then, beer ethanol was evaporated using a rotatory evaporator. Then, a solid-phase extraction (SPE) step was applied using HyperSepTM C18 500 mg cartridges (Thermo Scientific TM, Waltham, MA, USA). The cartridge was activated with 5 mL of methanol and rinsed with 15 mL of deionized water acidified with 37% hydrochloric acid (HCl). An amount of 10 mL of acidified beer was loaded into the cartridge and then rinsed with 15 mL of deionized water (not acidified). The elution of PC was carried out with 13 mL of 50% methanol acidified with acetic acid. The eluted fractions were evaporated until reaching approximately 1 mL of sample. The samples were stored at −20 °C until HPLC and LC-MS analysis.

##### Determination of Total Phenolic Content

The total phenolic content (TPC) was determined by employing a Folin–Ciocalteu assay (FC). An aliquot of beer before and after SPE-C18 was mixed with Folin–Ciocalteu reagent, water, and sodium carbonate solution (20%) [62]. The differences in the color of radical from light blue to dark blue were measured after 30 min at 750 nm using a UV/Vis microplate reader (Biotek PowerWave XS, New England, VT, USA). The TPC was quantified from the chlorogenic acid calibration curve (0–2 mg·mL^−1^, R^2^ = 0.9991). The TPC was calculated and expressed as mg of chlorogenic acid equivalents (CAE) per mL of beer. The measurements were performed in triplicate.

##### Identification and Characterization of Super Bock Beers

To proceed with the identification and characterization of PC compounds present in Super Bock beers, the four types of alcoholic beers tasted (Super Bock Original (SBO), Coruja American Amber Lager (AAL), Coruja India Pale Ale (IPA), and Coruja Hoppy Lager (HL) were studied. All beers were from the same batch as the tasted ones. All beers were produced in Portugal and were provided by the Super Bock Group.

##### High-Performance Liquid Chromatography (HPLC) Analysis

HPLC analysis was performed using an HPLC LC-4000 (JASCO) equipped with an Agilent C18 column (2.7 µm diameter and column dimensions of 4.6 × 250 mm), and detection was performed at 253, 280, 306, and 330 nm using a diode array detector (DAD). The gradient elution of analytes was carried out with 1% acetic acid in water (solvent A) and 1% acetic acid in acetonitrile (solvent B) at a constant flow rate of 0.5 mL/min, and the injection volume was 100 µL. The column temperature was set at 25 °C. A gradient elution protocol was applied as follows: 0 min, 5% B; 0–50 min, 18% B; 50–51 min, 21% B; 51–70 min, 30% B; 70–73 min, 50% B; 73–80 min, 75% B; 80–82 min, 100% B; 82–84 min, 100% B; 84–88 min, 100% B, 88–90 min, 5% B; and 90–105 min, 5% B. All samples obtained in Section 4.1 were injected directly for HPLC analysis.

##### Liquid Chromatography–Mass Spectrometry (LC-MS) Analysis

To identify the PCs present in each type of beer, all beer fractions obtained in Section 4.2.1 were analyzed with LC-MS using an Orbitrap Exploris Mass Spectrometer, in a negative and positive mode, for accurate mass measurements. The column, the solvents, and the method (gradient, detection wavelength, and flow rate) used for the LC-MS analysis were the same as those referred to previously for the HPLC analysis (Section High-Performance Liquid Chromatography (HPLC) Analysis). The mass spectra range was from *m*/*z* 100 to 2000, and the data analysis was achieved using Xcalibur software v2.0.7 (Thermo Fisher Scientific, Waltham, MA, USA).

### 4.3. Statistical Analysis

Statistical analysis of the phenolic content in each beer and the interaction between SPs and PCs were performed at least in triplicate in independent experiments. The mean values and standard error of the mean (SEM) were evaluated using analysis of variance (ANOVA) followed by Tukey’s multiple comparison test, and all statistical data were processed using GraphPad Prism version 8.0 for Windows (GraphPad Software, San Diego, CA. For PC content determination, values were considered statistically different at *p* < 0.0001, and for the interaction studies, at *p* < 0.05.

To assess the effect of SPs and PCs on astringent and bitterness perception and to evaluate the panel used, a multifactor ANOVA analysis (MFA) and sensory panel analysis, respectively, were performed using the XLSTAT version 2020.4.1.1020 software.

## 5. Conclusions

In this study, the astringency and bitterness of four different beers were evaluated by a sensory panel and was coupled to the study of the SP behavior and beer compound profile.

Overall, the findings indicate that beers with a higher PC content (HL and SBO) are more astringent and bitter than beers with a lower PC level (AAL and IPA). According to the correlation results, an increase in SP content under stimulation should reduce astringency and bitterness perception, in spite of which families of SPs account for the overall saliva content changes.

Ongoing studies are focused on studying the astringency of beers, focusing on the interaction with other oral constituents, namely oral epithelial cells, to unravel the mechanisms within the oral cavity that could contribute to this perception.

## Figures and Tables

**Table 1:** Sensory analysis of four different types of beer from Super Bock Group^®^, Hoppy Lager (HL), Super Bock Original (SBO), American Amber Lager (AAL), and India Pale Ale (IPA). Letters A and B represents astringency and bitterness, respectively.

Panelist	HL		SBO		AAL		IPA	
	A	B	A	B	A	B	A	B
P1	1 ± 0.01	3 ± 0.07	2 ± 0.00	2 ± 0.01	3 ± 0.03	4 ± 0.20	5 ± 0.07	3 ± 0.01
P2	1 ± 0.01	1 ± 0.17	1 ± 0.09	1 ± 0.07	2 ± 0.27	1 ± 0.31	3 ± 0.17	2 ± 0.17
P3	1 ± 0.01	4 ± 0.35	3 ± 0.14	1 ± 0.07	5 ± 0.23	2 ± 0.05	4 ± 0.01	3 ± 0.01
P4	2 ± 0.07	2 ± 0.01	3 ± 0.14	3 ± 0.17	4 ± 0.02	4 ± 0.20	4 ± 0.01	2 ± 0.17
P5	1 ± 0.01	1 ± 0.17	2 ± 0.00	2 ± 0.01	4 ± 0.02	3 ± 0.01	5 ± 0.07	4 ± 0.07
P6	2 ± 0.07	3 ± 0.07	2 ± 0.00	2 ± 0.01	4 ± 0.02	3 ± 0.01	5 ± 0.07	4 ± 0.07
P7	1 ± 0.01	2 ± 0.01	1 ± 0.09	1 ± 0.07	4 ± 0.02	2 ± 0.05	4 ± 0.01	3 ± 0.01
P8	1 ± 0.01	2 ± 0.01	2 ± 0.01	1 ± 0.07	3 ± 0.03	3 ± 0.01	4 ± 0.01	4 ± 0.07
P9	1 ± 0.01	2 ± 0.01	1 ± 0.09	3 ± 0.17	3 ± 0.03	2 ± 0.05	4 ± 0.01	4 ± 0.07
mean ± SEM	1.2 ^a^ ± 0.1	2.2 ^a,b^ ± 0.3	1.9 ^a,b^ ± 0.3	1.8 ^a^ ± 0.3	3.6 ^b^ ± 0.3	2.7 ^a,b^ ± 0.3	4.2 ^b^ ± 0.2	3.2 ^b^ ± 0.3

The numerical data represent the panelists’ classification according to their taste perception from low to high (1 to 5) astringency and bitterness. Values within the table are the score attributed to astringency and bitterness ± variance. Values in the last line are means of the panelists scores ± standard error of the mean (SEM). Values with different superscripts (a and b) are statistically different at *p* < 0.001. The analysis was performed comparing the different beers in the same sensory attribute (e.g., astringency in HL compared to the astringency in the remaining beers).

**Table 2:** Total phenolic content (TPC) of the analyzed beers, after Solid-Phase Extraction (SPE).

Beers	Folin–Ciocalteu (mg·mL^−1^ of CAE)
	After SPE
HL	0.461 ^a^ ± 0.002
SBO	0.554 ^b^ ± 0.003
AAL	0.897 ^c^ ± 0.003
IPA	0.966 ^d^ ± 0.004

Values are means of three measurements ± standard error of the mean (SEM). Values are presented by means of mg chlorogenic acid equivalents (CAE) per mL of beer. Values with different superscripts (a, b, c, and d) are statistically different at *p* < 0.001.

**Table 3:** Phenolic compounds present in the analyzed beers and their taste quality description. “A/B” description indicates astringency (A) and bitterness (B) perception.

		Beer				Description
Classification	Name	HL	SBO	AAL	IPA	A/B
Flavanols	(-)-Epicatechin	x	x	x	x	B [33,34]
	Gallo-catechin	-	x	x	-	A, B [36]
Flavonoids	6-Prenylnaringenin	x	x	x	x	B [37]
	8-Prenylnaringenin	x	x	x	x	B [38,39]
	Apiin	-	x	x	x	n.d.
	Naringenin	-	-	x	x	B [36,37]
	Quercetin	-	x	x	x	A, B [40]
Chalcone	Xanthohumol	x	x	x	-	B [41]
Phenolic acids	2-Hydroxycinnamic acid	x	x	x	x	A, B [42,43]
	4-Hydroxycinnamic acid	-	x	x	x	n.d.
	3-O-Caffeoylquinic acid (chlorogenic acid)	-	-	x	x	n.d.
	4-Feruloylquinic acid	-	-	x	x	n.d.
	4-O-Caffeoylquinic acid	x	-	x	x	n.d
	5-O-Caffeoylquinic acid (neochlorogenic acid)	-	-	x	x	n.d.
	Feruloylputrescine	-	-	x	-	n.d.
Stilbene	Resveratrol	x	x	x	x	B [42,43,44]
Other phenolic compounds	4-Methylumbelliferyl glucuronide		x	x	x	n.d.
	7-Hydroxycoumarin	x	x	x	x	B [45]
	Coumarin 106	-	x	x	x	B [45]
	Coumaroylagmatine	-	x	x	x	n.d.
	Di-caffeoyl spermidine	-	-	x	-	n.d.
	Diferuloylputrescine	x	x	x	x	n.d.
	Esculin	x	x	x	x	B [46]
	Feruloylagmatine	x	x	x	x	n.d.
	Lupulone	x	-	x	x	B [47]
	Met-coumaroylagmatine hexoside	x	x	x	x	n.d.
	Sinapoylagmatine	x	-	x	x	n.d.

“n.d.” description specifies that the indicated compounds were not determined neither as astringent and/or bitter. The “x” letter and the “-” symbol indicate that the referred compound was present and absent, respectively, in the corresponding beer.

**Table 4:** Nonphenolic compounds present in the studied beers and their taste quality description. “A/B” description indicates astringency (A) and bitterness (B) perception.

		Beer				Taste Quality
Classification	Name	HL	SBO	AAL	IPA	A/B
Amino acid	Leucine	x	-	x	x	B [5]
	L-Phenylalanine	x	x	x	x	B [45,46]
	L-Tryptophan	x	x	x	x	A, B [5]
	Tyrosine	x	-	x	x	B [5]
Peptide	Leucylvaline	x	-	x	x	n.d.
	Pyroglutamyl-Isoleucine	x	x	x	x	n.d.
	Cyclo(proline-leucine)	x	x	x	x	n.d.
	Glutamyltyrosine	x	x	x	x	n.d.
Alkaloid	4-Hydroxyquinoline	-	x	x	-	n.d.
	8-Hydroxyquinoline	x	x	x	x	B [48]
	Caffeine	x	x	x	x	B [5,49]
	Hordenine	x	x	x	x	B [50]
Benzaldehyde	Vanillin	-	x	x	x	* activates TAS2R14, TAS2R20 and TAS2R39 [51]
Carboxylic acid	Citric acid	x	x	x	x	A [52]
Fatty acid	Linoleic acid	-	x	x	x	A, B [53]

“n.d.” description specifies that the indicated compounds were not determined neither as astringent and/or bitter. The “x” letter and the “-” symbol indicate that the referred compound was present and absent, respectively, in the corresponding beer. “*” symbol indicates an additional information regarding the respective compound.

**Table 5:** Beer compounds’ variability within the studied beers. The peak area value was determined by the integration of each corresponding compound peak in each beer in the TIC chromatogram. (A) Astringent and (B) bitter attributes.

		Chromatographic Peak Areas (×10^9^)			
Compound		HL	SBO	AAL	IPA
14	Tyrosine (B)	0.76	n.d.	1.57	0.87
23	L-phenylalanine (B)	1.03	1.29	3.34	1.95
38	L-tryptophan (A and B)	2.00	3.93	4.50	3.52
50	(-)-epicatechin (B)	3.78	3.65	6.27	5.10
56	8-prenylnaringenin (B)	3.93	1.40	1.21	1.90
58	Quercetin (A and B)	n.d.	0.68	0.76	0.84
62	Lupulone (B)	3.02	n.d.	3.54	5.20

The remaining compounds do not have any description according to taste modality, as astringent and/or bitter. “n.d.” description specifies that the indicated compounds were not determined in the TIC profile in the corresponding beer.

## Data Availability

The data presented in this study are available on request from the corresponding authors.

## Supplementary Materials

The following supporting information can be downloaded at: https://www.mdpi.com/article/10.3390/molecules28062522/s1, Table S1: Phenolic and nonphenolic compounds “tentatively” identified in four Super Bock beers, after Solid-Phase Extraction (SPE), using LC-MS analysis; Figure S1: Total ion current (TIC) profile of each beer in negative and positive mode, obtained using LC-MS analysis; Figure S2: Proline-rich protein (PRPs; A. bPRPs, B. gPRPs, and C. as aPRPs) family behavior after intake of each beer; Figure S3: (A) Statherin/P-B peptide and (B) Cystatin behavior after intake of each beer.

## Sample Availability

Samples of the compounds are available from the authors.
