# Supplementary material for: Interactions between Beer Compounds and Human Salivary Proteins: Insights toward Astringency and Bitterness Perception
Source: Molecules. 2023 Mar 9;28(6):2522. doi: 10.3390/molecules28062522 (PMC10053927; doi:10.3390/molecules28062522)
Supplement: Supplementary file 1 [file molecules-28-02522-s001.zip › molecules-2210353-supplementary.pdf]

Table S1. Phenolic and non-phenolic compounds "tentatively" identified in four Super Bock beers, after Solid Phase Extraction (SPE), by LC-MS analysis.

Compound	Name	t_R (min)	$[M-H]^-$	MS^2 (-) (m/z)	$[M-H]^+$	MS^2 (+) (m/z)
**1**	2-Hydroxycinnamic acid	4.86	162.84	162.84 (100)	n.d.	n.d.
**2**	Quinic acid or Scopolin	5.18	191.06	191.06 (100)	n.d.	n.d.
**3**	7-Hydroxycoumarin	5.53	160.84	160.84 (100)	n.d.	n.d.
**4**	4-Hydroxyquinoline	5.64	n.d.	n.d.	146.12	146 (100)
**5**	Adenine	5.71	n.d.	n.d.	136.06	136.06 (100)
**6**	8-Hydroxyquinoline	6.11	n.d.	n.d.	146.09	146 (100); 128.03 (11.12); 104.07 (15.80); 87.06 (50.43); 86.06 (33.67)
**7**	D-Sorbitol	6.33	n.d.	n.d.	189.08	189.08 (11.92); 129.05 (52.51); 111.04 (20.77); 99.04 (23.17); 85.03 (14.90); 69.03 (100)
**8**	Cis-resveratrol	6.71	n.d.	n.d.	229.15	229.15 (100); 142.09 (8.60); 70.07 (2.17)
**9**	Citric acid	6.76	191.02	191.02 (10.68); 111.01 (100); 87.01 (46.20); 86.03 (1.18); 85.03 (30.38)	n.d.	n.d.
**10**	Pipecolic acid	7.17	128.04	128.04 (100)	130.05	130.05 (28.32); 84.04 (100)
**11**	Linoleic acid	7.36	279.04	279.23 (1.73); 243.06 (100);111.02 (62.70); 110.02 (42.53)	n.d.	n.d.
**12**	Vanillin	7.54	151.03	151.03 (100); 108.02 (19.64)	153.04	153.04 (100); 136 (2.38); 110.03 (11.29)
**13**	Adenosine	7.73	n.d.	n.d.	136.06	314.09 (2.76); 136.06 (100)
**14**	Tyrosine	8.32	n.d.	n.d.	182.08	182.08 (4.70); 165.05 (50.33); 147.04 (20.02); 136.08 (100); 123.04 (34.31)
**15**	Leucine	9.05	n.d.	n.d.	132.1	132.10 (4.55); 87.10 (5.39); 86.10 (100)
**16**	Guanosine	9.5	282.08	282.08 (11.99); 150.04 (100)	284.1	152.06 (100)
**17**	Hordenine	10.92	n.d.	n.d.	166.12	166.12 (29.14); 122.07 (7.04); 121.06 (100)
**18**	Adenine dimer	12.44	n.d.	n.d.	282.12	136 (100)
**19**	3-*O*-Acetyldiosgenin trimer	12.85	n.d.	n.d.	1387.96	325.11 (100); 289.09 (9.45)
**20**	Uridine	13.13	n.d.	n.d.	245.1	181 .10 (100); 125.07 (3)
**21**	Beta-carboline	13.31	n.d.	n.d.	169.1	170.10 (8.73); 169 (100); 141 (4.75)
**22**	1-Methyladenosine	13.94	n.d.	n.d.	282.12	282.12 (6.32); 150.08 (100)
**23**	L-Phenylalanine	14.38	164.07	164.07 (60.97); 148.05 (7.91); 147.05 (100)	166.09	166.09 (4.25); 149.06 (1.80); 121.08 (7.44); 120.08 (100); 107.05 (1.63); 103.05 (1.61)
**24**	Xanthohumol	14.69	353.15	353.15 (10.08); 239.09 (10.50); 238.12 (22.80); 238.08 (100); 126.02 (36.85); 114.06 (55.46)	355.16	240.07 (9.72); 227.10 (10.33); 212.10 (10.68); 129.07 (30.47); 116.07 (100)
**25**	Vitamin B5	15.45	218.1	218.10 (25.49); 146.08 (100)	220.12	220.12 (60.57); 202.11 (100); 184.10 (82.70); 142.09 (24.50); 116.03 (51.60); 103.07 (30.05)
**26**	Coumarin 106	16.28	n.d.	n.d.	282.19	283.20 (13.84); 282.19 (100); 195.11 (64.36)
**27**	Quercetin dimer	17.15	602.13	602.12 (42.14); 490.10 (36.49); 362.05 (42.01); 225.02 (65.03); 111.02 (100)	604.14	506.60 (0.43); 357.15 (1.78); 227.03 (0.54); 152.06 (100)
**28**	Adenosine (267)	17.32	326.14	282.11 (54.75); 238.12 (55.48); 167.05 (100); 114.06 (40.33)	n.d.	n.d.
**29**	Apiin	18.67	563.1	563.10 (13.21); 451.08 (11.85); 225.02 (10.86); 111.02 (100)	n.d.	n.d.
**30**	Caffeine	19.18	n.d.	n.d.	195.11	195.11 (100)
**31**	L-Tryptophan	19.49	n.d.	n.d.	227.18	227.18 (100); 209.16 (21.07); 114.09 (14.48)

**32**	Gallo-catechin	20.46	613.18	613.18 (3.50); 289.07 (100); 245.08 (15.88); 137.02 (16.94)	n.d.	n.d.
**33**	Resveratrol	21.67	227.1	227.10 (49.76); 183.11 (100)	229.12	229.12 (5.26); 183.11 (100); 118.09 (81.35); 116.07 (52.74)
**34**	5-*O*-Caffeoylquinic acid (neochlorogenic acid)	21.93	353.09	353.09 (1.60); 191.06 (100); 179.04 (75.26); 135.05 (19.31)	n.d.	n.d.
**35**	Soyasapogenol A	22.1	n.d.	n.d.	439.22	439.22 (100)); 277.17 (22.46); 260.14 (15.48); 147.04 (75.39)
**36**	4-Methylumbelliferyl glucuronide	22.37	351.13	207.09 (100); 99.05 (99.40)	n.d.	n.d.
**37**	Adenine	24.21	n.d.	n.d.	136.06	298.10 (1.13); 136.06 (100)
**38**	L-Tryptophan	25.19	n.d.	n.d.	188.07	188.07 (29.78); 146.06 (100); 144.08 (20.86)
**39**	L-Glutamine dimer	25.61	291.1	291.10 (22.94); 247.11 (17.84); 141.07 (10.47); 127.05 (100); 119.05 (10.61); 109.04 (13.36)	n.d.	n.d.
**40**	L-Glutamine	25.92	n.d.	n.d.	146.06	146.06 (100)
**41**	Naringenin	28.98	n.d.	n.d.	273.13	273.13 (79.26); 255.12 (97.56); 213.10 (15.57); 147.04 (80.69); 127.10 (100); 110.07 (22.07)
**42**	Feruloylputrescine	30.7	n.d.	n.d.	265.15	265.15 (3.79); 248.13 (5.65); 177.05 (100)
**43**	3-O-Caffeoylquinic acid (chlorogenic acid)	31.79	353.09	191.06 (100)	n.d.	n.d.
**44**	4-O-Caffeoylquinic acid	34.7	353.09	353.09 (1.12); 191.06 (47.02); 179.04 (80.66); 173.05 (100); 135.05 (26.50)	n.d.	n.d.
**45**	Isoxanthohumol	35.46	353.18	353.09 (29.68); 335.17 (18.48); 207.06 (100); 147.12 (12.78)	n.d.	n.d.
**46**	Met-coumaroylagmatine hexoside	35.9	n.d.	n.d.	453.22	453.22 (25.39); 425.22 (13.96); 372.19 (11.57); 291.16 (100); 157.11 (9.09); 145.05 (5.94); 127.04 (8.56)
**47**	Coumaroylagmatine	36.28	275.15	275.15 (11.22); 233.13 (20.66); 119.05 (100)	277.17	277.17 (91.19); 260.14 (36.35); 2218.12 (20.04); 147.04 (100); 114.10 (43.36)
**48**	Feruloylagmatine	40.58	n.d.	n.d.	307.16	307.18 (61.19); 290.15 (15.99); 177.05 (100); 114.10 (19.74)
**49**	Pyroglutamyl-Isoleucine	41.32	241.12	241.12 (58.76); 197.13 (100); 141.10 (9.04)	243.14	243.14 (4.85); 197.13 (53.10); 132.10 (100)
**50**	(-)-Epicatechin	41.65	n.d.	n.d.	292.15	291.06 (100); 283.15 (17.63); 265.08 (27.46); 173.10 (30.69); 147.12 (98.63); 113.07 (38.67)
**51**	Sinapoylagmatine	42.72	n.d.	n.d.	337.19	337.19 (44.28); 320 .16 (8.33); 207.07 (100); 114.10 (12.74)
**52**	Cyclo(proline-leucine)	43.6	n.d.	n.d.	211.14	211.14 (100)
**53**	Di-caffeoyl spermidine	43.86	468.25	468.25 (62.41); 424.26 (29.44); 278.19 (27.68); 244.13 (13.91); 240.14 (12.47); 145.06 (99.22); 127.05 (100)	470.26	246.15 (12.84); 147.08 (100); 130.05 (51.26)
**54**	4-Hydroxycinnamic acid (4-Coumaric acid)	51.6	163.04	163.04 (14.43); 119.05 (100)	n.d.	n.d.
**55**	Esculin or 6-Prenylnaringenin	53.67	338.17	338.17 (11.12); 294.18 (29.15); 223.11 (89.55); 180.10 (11.64); 114.06 (100)	n.d.	n.d.
**56**	8-Prenylnaringenin	54.24	338.17	338.17 (11.12); 294.18 (29.15); 223.11 (89.55); 180.10 (11.64); 114.06 (100)	340.19	197.13 (22.04); 116.07 (100); 86.10 (10.32)
**57**	Leucylvaline	54.74	n.d.	n.d.	344.25	231.17 (11.06); 185.16 (76.77); 86.10 (100); 72.08 (36.94)
**58**	Quercetin	55.3	n.d.	n.d.	303.16	303.16 (66.58); 291.06 (57.10); 273.64 (42.62); 265.09 (35.50); 211.12 (100); 185.14 (43.62); 114.07 (32.63)
**59**	Diferuloylputrescine	55.91	n.d.	n.d.	342.2	291.07 (51.08); 265.09 (100); 115.09 (14.81)
**60**	Glutamyltyrosine	56.21	n.d.	n.d.	311.1	311.10 (100)
**61**	4-Feruloylquinic acid	58.15	n.d.	n.d.	366.2	367.21 (0.89); 173.13 (100)
**62**	Lupulone	61.91	413.22	395.21 (20.88); 338.17 (18.79); 337.17 (100); 195.07 (16.07)	n.d.	n.d.

n.d. = not detected

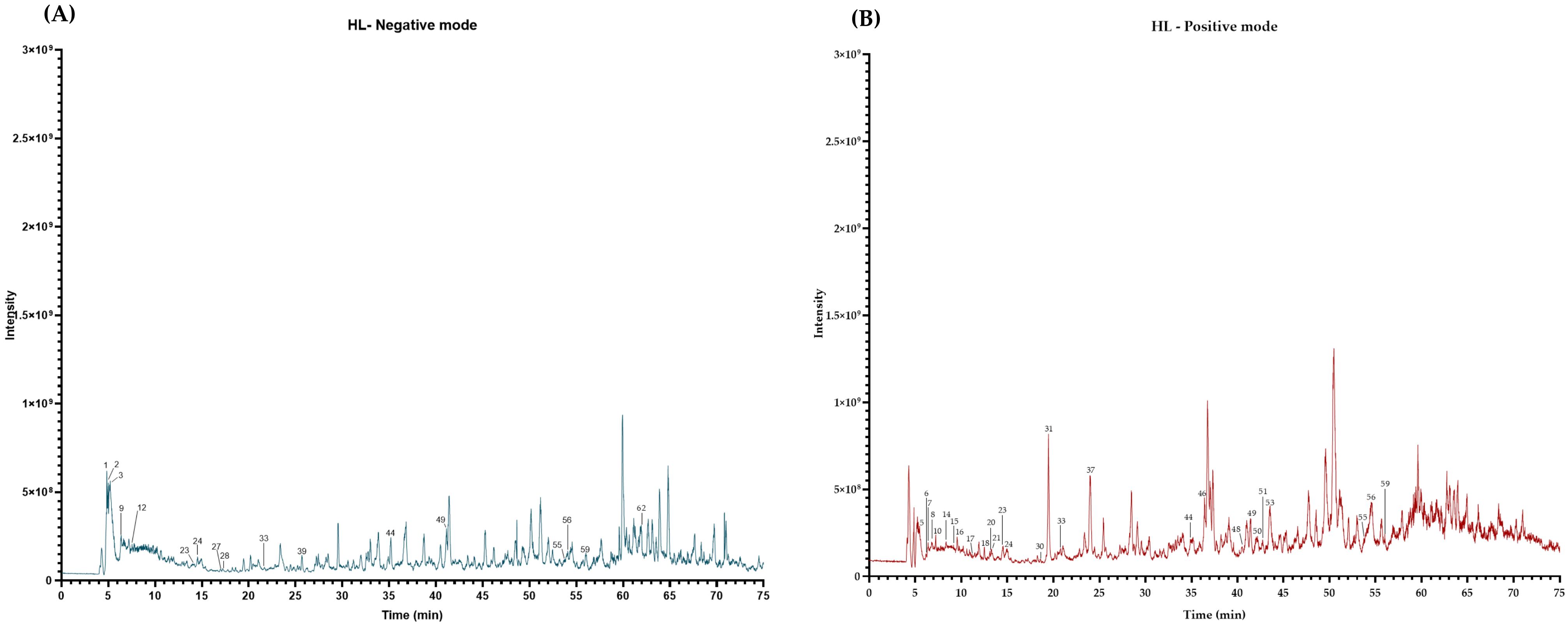

Figure S1. Total ion current (TIC) profile of Hoppy Lager (HL) beer in (A) negative and (B) positive mode, obtained by LC-MS analysis. Superscript numbers represent the compounds putatively identified in this beer.

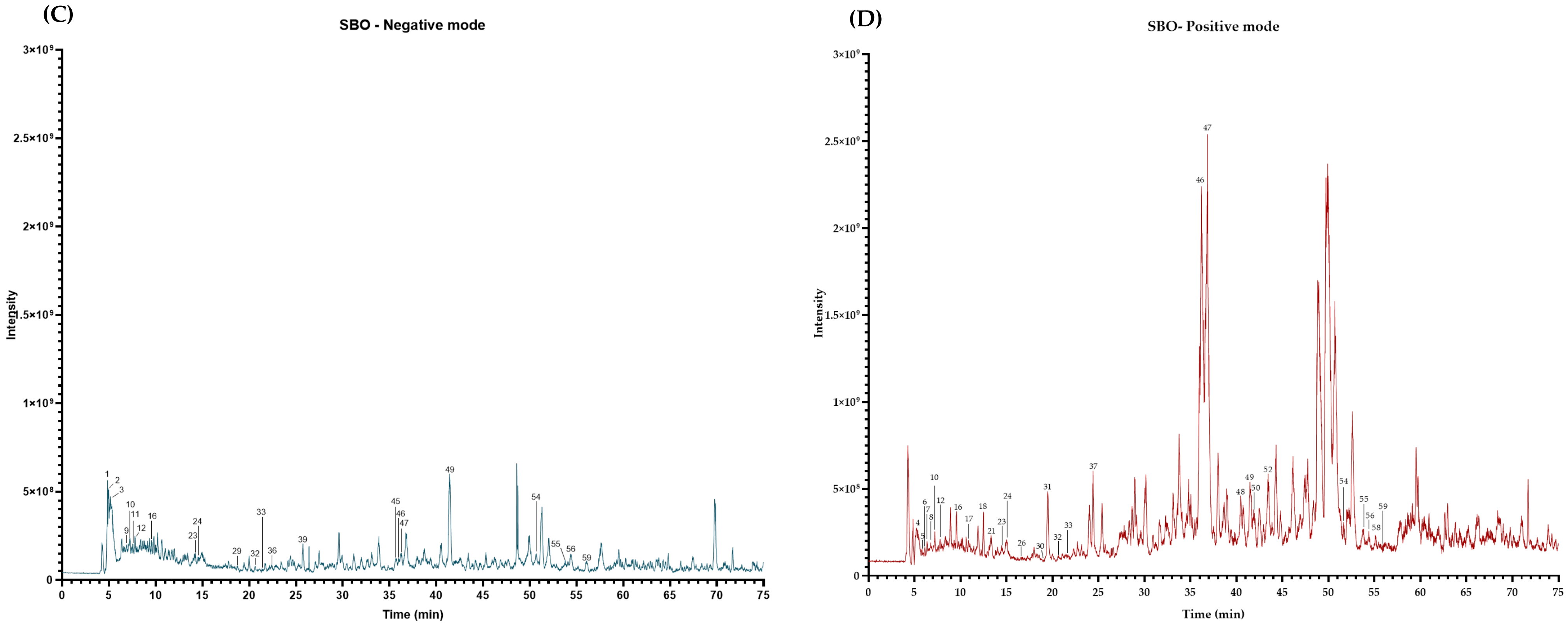

Figure S1. Total ion current (TIC) profile of Super Bock Original (SBO) beer in (C) negative and (D) positive mode, obtained by LC-MS analysis. Superscript numbers represent the compounds putatively identified in this beer.

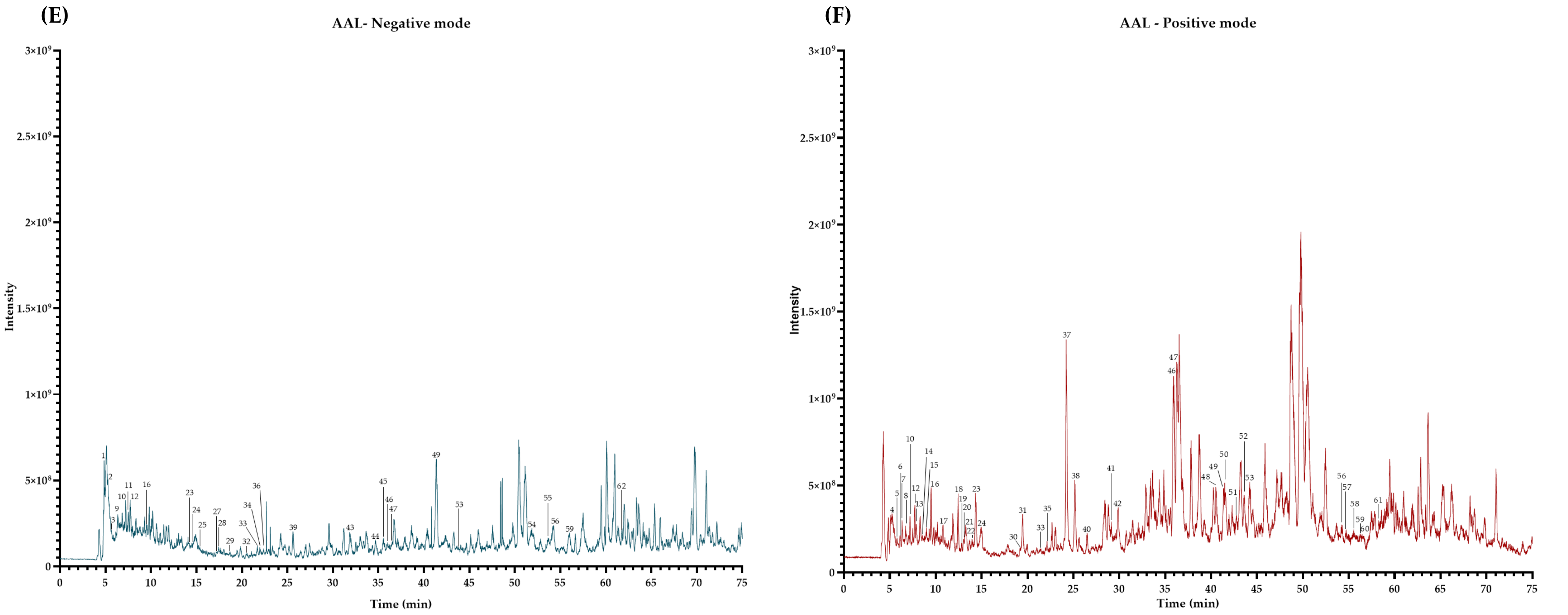

Figure S1. Total ion current (TIC) profile of American Amber Lager (AAL) beer in (E) negative and (F) positive mode, obtained by LC-MS analysis. Superscript numbers represent the compounds putatively identified in this beer.

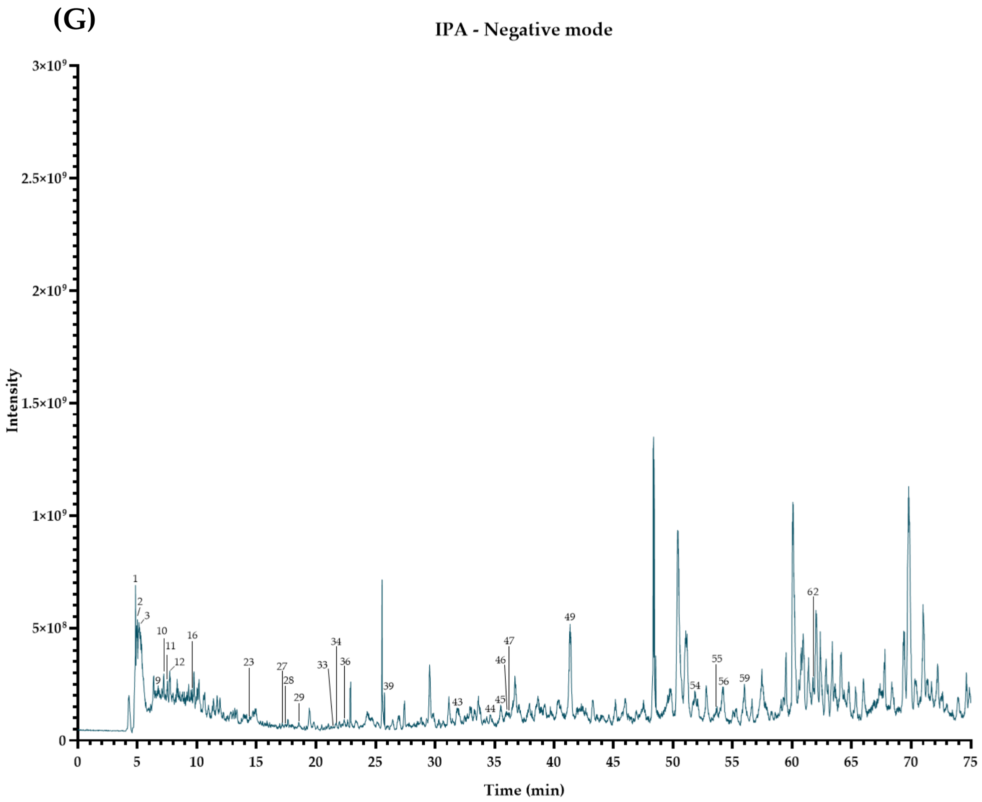

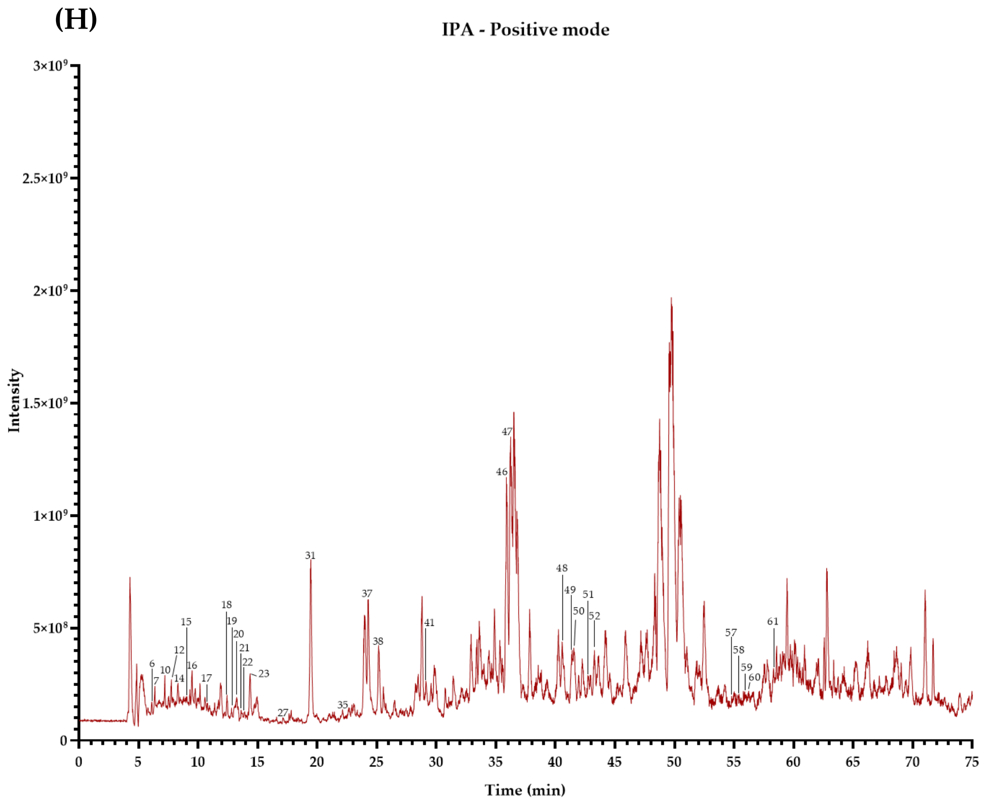

Figure S1. Total ion current (TIC) profile of India Pale Ale (IPA) beer in (G) negative and (H) positive mode, obtained by LC-MS analysis. Superscript numbers represent the compounds putatively identified in this beer.

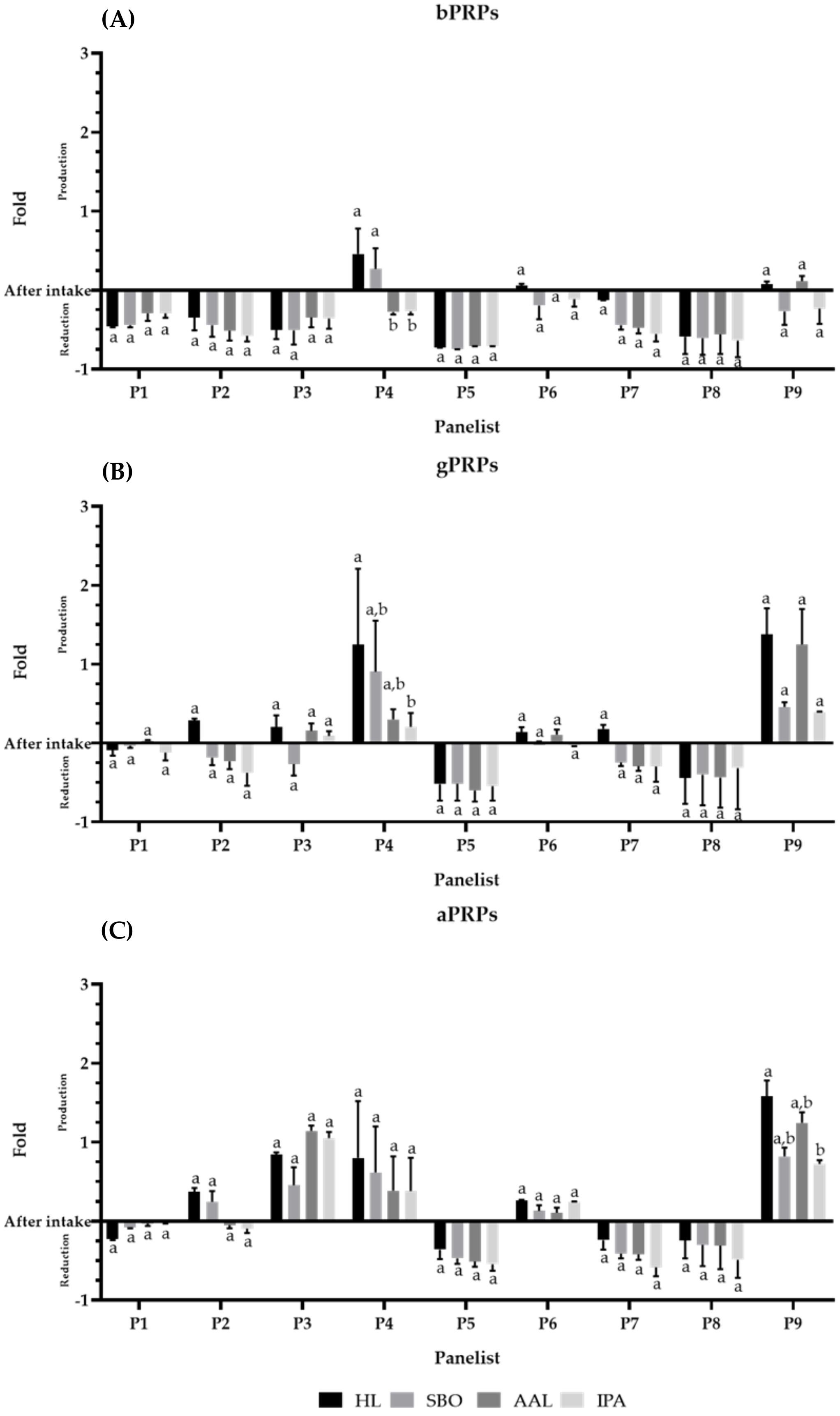

Figure S2. Proline-rich proteins (PRPs; A. bPRPs, B. gPRPs, and C. as aPRPs) family behavior after intake of each beer, where Coruja Hoppy Lager (HL), Super Bock Original (SBO), Coruja American Amber Lager (AAL), and Coruja India Pale Ale (IPA), respectively. The interaction between the PRPs and PC compounds is presented by the fold of each PRP precipitated, for each panelist, during beer sensorial analysis. Fold represents how many times occurs SP reduction or production regarding to the previous beer intake of saliva collection for each panelist. Values with different letters within each panelist are significantly different at $p<0.05$.

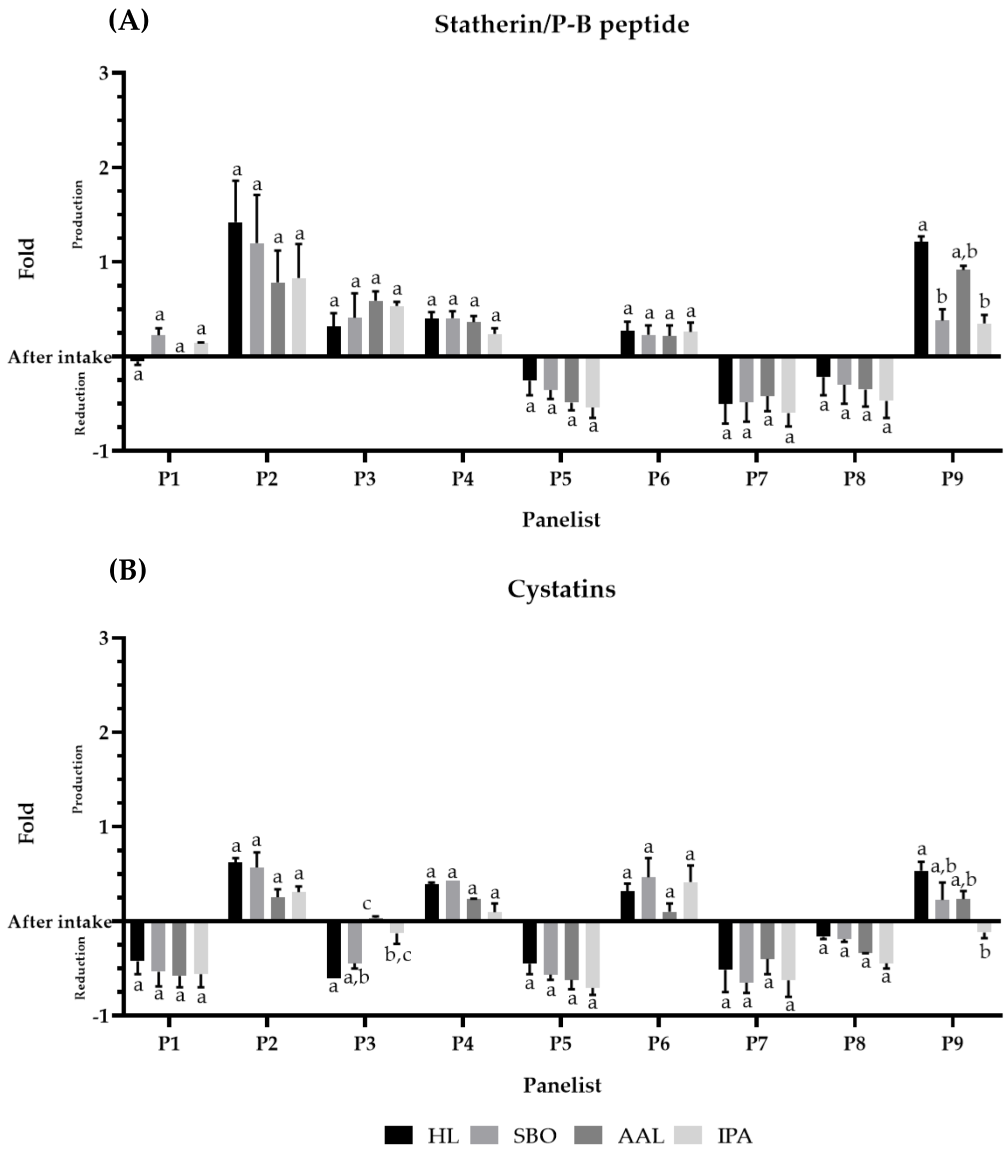

Figure S3. (A) Statherin / P-B peptide and (B) Cystatins behavior after intake of each beer, where Coruja Hoppy Lager (HL), Super Bock Original (SBO), Coruja American Amber Lager (AAL), and Coruja India Pale Ale (IPA), respectively. The interaction between these SP and PC compounds is presented by the fold of each PRP precipitated, for each panelist, during beer sensorial analysis. Fold represents how many times occurs SP reduction or production regarding to the previous beer intake of saliva collection for each panelist. Values with different letters within each panelist are significantly different at $p<0.05$.
